# Antibiofilm Potential and Exoenzyme Inhibition by *Elattaria cardamomum* Essential Oil in *Candida* spp. Strains

**DOI:** 10.3390/life12111756

**Published:** 2022-11-01

**Authors:** Emira Noumi, Ghada Salamah Alshammari, Tarek Zmantar, Abdulrahman S. Bazaid, Khulood Fahad Alabbosh, Abdelbaset Mohamed Elasbali, Waleed Abu Al-Soud, Sami Ghazi Alrashidi, Mejdi Snoussi

**Affiliations:** 1 Department of Biology, College of Science, University of Hail, Hail 2440, Saudi Arabia; 2 Laboratory of Analysis, Treatment and Valorization of Environmental and Product Pollutants, Faculty of Pharmacy of Monastir, Avicenne Street, Monastir 5000, Tunisia; 3 Department of Medical Laboratory Science, College of Applied Medical Sciences, University of Hail, Hail 55476, Saudi Arabia; 4 Department of Clinical Laboratory Science, College of Applied Sciences-Qurayyat, Jouf University, Sakaka 72388, Saudi Arabia; 5 Department of Clinical Laboratory Science, College of Applied Sciences-Sakaka, Jouf University, Sakaka 72388, Saudi Arabia; 6 Immunology Laboratory, King Salman Specialist Hospital, Hail 55471, Saudi Arabia; 7 Laboratory of Genetics, Biodiversity and Valorisation of Bioresources, High Institute of Biotechnology, University of Monastir, Monastir 5000, Tunisia

**Keywords:** *Candida* species, exoenzymes, biofilm inhibition, virulence factors, *Elettaria cardamomum*

## Abstract

Fungal infections caused by *Candida* species have attracted great interest due to their resistance to commercial antifungal agents. Essential oils from aromatic and medicinal plants have many bioactive compounds that are known for their important biological activities, mainly their antimicrobial effects. In the present study, we aimed to evaluate the antifungal ability of *Elettaria cardamomum* essential oil (EO) against different clinical *Candida* isolates. Then, we investigated the anti-phospholipase, anti-protease, and anti-biofilm activity of *E. cardamomum* EO against the selected isolates. Twenty-four *Candida* strains (clinical and reference) were tested for virulence factors such as biofilm formation, protease, and phospholipase activity. The minimum inhibitory (MIC) and fungicidal (MFC) concentrations of *E. cardamomum* were determined, and their effects were tested against all *Candida* strains. Our results revealed that *E. cardamomum* EO was rich in α-terpinyl acetate (56.5%), limonene (12.6%), and mentha-2.4(8)-diene (7.65%). The tested EO showed activity against all tested *Candida* strains in their planktonic form and against exoenzymes and biofilm production. Based on our findings, we promote the use of *E. cardamomum* EO as a treatment against clinical *Candida* isolates active on the virulence factors of this fungus.

## 1. Introduction

Fungal infections with increasing resistance to conventional therapies are a growing concern. *Candida* species are major agents of human infections. *Candida albicans* is a major opportunistic yeast responsible for mucosal and invasive infections [1,2]. Today, *Candida* species are emerging as major agents of hospital-acquired infections. These species are ranked as the third- or fourth-most isolated bloodstream pathogens. Although *C. albicans* is recognized as the predominant etiological agent of candidiasis, other *Candida* species such as *C. krusei*, *C. glabrata*, *C. lusitaniae*, and *C. dubliniensis*, which tend to be less susceptible to common antifungal drugs, have also emerged as substantial opportunistic pathogens [1,2]. Importantly, yeasts (mainly *C. albicans*) are the third leading cause of catheter-related infections. *C. dubliniensis* isolates have usually been recovered from symptomatic HIV-infected individuals and are unidentifiable as any known *Candida* species using conventional criteria [2]. Biofilm-associated infections are therefore difficult to treat because of their decreased susceptibility to antimicrobial therapy. In fact, resistance has been reported to increase 1000-fold under some conditions. In the case of *C. albicans* biofilms, our group and others have reported that they are up to 4000 times more resistant to fluconazole when compared with planktonic, free-floating cells [1].

*Candida* strains have developed resistance to many antifungal agents, and as a result, immense clinical problems have emerged in the treatment of these diseases [3]. The resistance of organisms has increased due to the indiscriminate use of commercial antifungal drugs, commonly used for the treatment of infectious diseases. These fungi possess various virulence factors (like exopolysaccharides production), phospholipase and protease production, hemolysins secretion, surface hydrophobicity, phenotypic switching, biofilm formation, and adherence to epithelial cells [4,5], which promote the dissemination of infections in susceptible hosts. To infect hosts, *Candida* yeast cells adhere to host cells using adhesins [6]. Biofilms are structured communities [7] that enable fungus attachment to biotic or abiotic surfaces [8], including medical devices [9]. To prevent *Candida* infection, all these virulence factors can be targeted. Recently, research toward new antifungal agents has aimed at the selective targeting of virulence mechanisms as opposed to the killing of the pathogen, which may increase the selection pressure for development of drug resistance [10].

The emergence and spread of antifungal resistance are growing global threats that have aroused a global interest in limiting antifungal use to treat human candidiasis. Since ancient times, traditional healing systems provided some herbal remedies to treat human diseases. Although phytotherapeutic remedies are available and are used, evidence of their efficacy is currently very limited but is nevertheless still necessary. Researchers have been encouraged to seek new antimicrobial compounds from various sources, including medicinal plants [3]. From natural and medicinal plants, we notice that *Elettaria cardamomum* (green cardamom) has been used as an aromatic, diuretic, and stimulant agent to treat cardiovascular and gastrointestinal disorders. This herb is used to prevent and treat asthma, bronchitis, kidney stones, and urinary tract disorders [11]. Some studies have demonstrated the antimicrobial effect of this plant [10,11,12]. Many studies have been made on the chemical and antimicrobial properties of *E. cardamomum* [13,14] against *Staphylococcus aureus*, *Bacillus cereus*, *Pseudomonas aeruginosa*, and *Escherichia coli* [15].

However, the present study focuses on the antifungal activity of *E. cardamomum* essential oil (EO) against several *Candida* strains. The objective of our work was to study the virulence factors and biofilm formation of *Candida* strains from Hail hospitals and to validate the anti-*Candida* (enzymes production, planktonic, and biofilm forms) potential of *E. cardamomum* EO. The results obtained from the current project confirm the therapeutic properties of the selected EO and encourage its therapeutic use as a anti-phospholipase and anti-protease agent.

## 2. Materials and Methods

### 2.1. Patients, Strains, Media, and Growth Conditions 

This study was conducted following the Ethics Committee at Hail Affairs (reference: H-08-L-074). Patient privacy and confidentiality of data were maintained anonymously in accordance with The Declaration of Helsinki. The subjects of the study were twenty-four (24) patients from Hail region, Saudi Arabia with cutaneous infections. A collection of reference and clinical *Candida* strains was used during this study. Five *Candida* strains provided from American Type Collection Culture, *Candida utilis* ATCC 9255 (A1), *Candida guillermondii* ATCC 6260 (A4), *Candida tropicalis* ATCC 1362 (A8), *Candida albicans* ATCC 10231 (A14), *Candida albicans* ATCC 20402 (A15), and *Saccharomyces cerevisiae* ATCC 20407 (A9), were used as control. Thirteen *C. albicans* strains isolated from different medical services (C1, C2, C3, C4, C5, 104, 104W, 108, 109, 113, 124, 126, and 118) were the subjects of this work. Samples were collected from the patients with a sterile cotton swab (Nippon Menbo, Tokyo, Japan), which were immediately cultured on Sabouraud chloramphenicol agar for 24–48 h at 35 °C.

### 2.2. Phospholipase Assay 

Production of phospholipase by *Candida* strains was determined as described by Noumi et al. [16]. Cultures were transferred onto Sabouraud Chloramphenicol agar plates and incubated at 37 °C for 48 h for enzymatic tests. Following incubation, 1.5 mL of the yeast culture was centrifuged at 2000× *g* for 5 min. The pellets obtained were washed twice by resuspension in PBS and centrifuged under the same conditions to remove residual culture medium. One microliter (1 µL) of the suspension was then plated in duplicate in phospholipase agar medium [16]. The inoculated plates were incubated at 37 °C for 4 days. The presence of phospholipase was determined by the formation of an opaque zone around the yeast colonies, and it was measured by calculating the ratio of colony diameter to colony diameter with area of precipitation. The value of Pz is inversely proportional to the nature of the activity. Precipitation zone (Pz) reflecting the enzymatic activity was interpreted as phospholipase negative (Pz = 1), phospholipase positive (Pz > 0.63), and phospholipase very strong (Pz < 0.63).

### 2.3. Proteinase Assay

Determination of proteinase production was performed according to the method described by Noumi et al. [16]. The test medium consisted of agar plates containing bovine serum albumin (BSA, pH 3.5). Each strain was inoculated in triplicate, and the plates were incubated at 37 °C for 7 days. The presence of proteinase was determined by the formation of a transparent halo around the yeast colonies. Proteinase activity was measured by calculating the ratio of colony diameter to colony diameter with area of precipitation. The value of Pz is inversely proportional to the nature of the activity. The Pz coefficients of the *Candida* strains analyzed were grouped into 4 classes: very low activity (0.9 < Pz < 1), low activity (0.89 < Pz < 0.80), high activity (0.79 < Pz < 0.70), and very high activity (Pz < 0.69).

### 2.4. Qualitative Detection of Exopolysaccharide Production 

#### 2.4.1. Safranin Method 

Slime production was determined using the safranin method as described by Davenport et al. [17]. *Candida* isolates were inoculated into a tube containing 10 mL of Sabouraud broth supplemented with 8% of glucose. The tubes were incubated at 35 °C for 24 h and examined for the presence of a viscid slime layer after removing the cell suspension colored for 30 min with safranin. Slime production by each isolate was scored as negative, weak (+), moderate (++), or strong (+++).

#### 2.4.2. Detection of Slime Production by the Congo Red Agar (CRA) Method 

The slime-producing ability of *Candida* strains was tested according to the protocol described by Noumi et al. [18]. All isolates were cultured on Congo red agar plates. Plates were incubated at 37 °C for 24 h under aerobic conditions and subsequently left overnight at room temperature. After incubation, black colonies were interpreted as slime-positive and unpigmented colonies (white, pinkish red…) were interpreted as slime-negative strains.

### 2.5. Biofilm Formation on Polystyrene 

In the present study, biofilms were produced on commercially available presterilized polystyrene flat-bottom 96-well microtiter plates (Iwaki, Tokyo, Japan) for 48 h on yeast nitrogen-base medium. Batches of medium were inoculated with overnight yeast cultures and incubated at 37 °C in an orbital shaker operating at 150 rpm. Cells were harvested after 24 h (stationary growth phase), washed once with phosphate-buffered saline (PBS, pH 7.2), and standardized to a density at 10^7^ cells/mL. One hundred microliters of a standardized cell suspension (10^7^ cells/mL) was transferred to each well of a microtiter plate, and the plate was incubated for 48 h at 37 °C to allow yeasts to adhere to the well surfaces for 90 min. 

As negative controls, three wells of each plate were handled in an identical way, except that no *Candida* suspensions were added. Following the adhesion phase, non-adherent cells were removed from the wells by being gently washed twice with 200 μL ml PBS. One hundred microliter of yeast nitrogen base medium was transferred into each washed well, and the plates were incubated at 37 °C in a shaker at 75 rpm. 

Adherent biofilm was fixed with 95% ethanol and was stained with 100 μL of 1% of crystal violet (Merck, Lyon, France) for 5 min. Then, unbound crystal violet was removed, and the wells were washed three times with sterile distilled water. The water was then absorbed, and the microtiter plate was air-dried for 30 min. Then, the biofilm was dissolved into acetic acid (33%). Next, 125 μL from each well was transferred to a 96-well microtiter plate, and the OD at 570 nm was measured. Biofilm formation was categorized as highly positive (OD_570_ ≥ 1), low-grade positive (0.1 ≤ OD_570_ < 1), or negative (OD_570_ < 0.1) [19].

### 2.6. Plant Material and Extraction of Essential Oil

*Elettaria cardamomum* (fruits/seeds) were freshly purchased from a local market (Nabeul, Tunisia) in December 2021. The species was identified by Prof. Abderrezak Smaoui, an expert botanist from the Laboratory of Extremophile Plants, Biotechnology Centre of Borj-Cedria, University of Tunis El Manar, Hammam-Lif, Tunisia; a voucher specimens number NE-GC-001 was attributed to this sample.

The green cardamom was ground to fine powder using a Mettler AE 200 (Dangoumau-type) grinder apparatus (size 20 micrometer). Powdered cardamom (100 g) was subjected to volatile oil extraction for 3 h with 500 mL distilled water using a Clevenger-type apparatus, according to the European Pharmacopoeia (1975) [20]. The obtained oil was dried over anhydrous sodium sulphate and stored in sealed glass vials in a refrigerator at 4 °C prior to analysis.

### 2.7. Composition of Elattaria Cardamomum Essential Oil

Gas chromatography with electron-impact mass spectrometry (GC-EIMS) analyses were performed according to the protocols previously described by Davies (1990) and Adams (1995) based on the calculation of the retention times using the n-alkanes series (C8–C23) [21,22].

### 2.8. Effect of Elattaria Cardamomum Essential Oil on Candida strains 

#### 2.8.1. Disk Diffusion Assay

The anti-*Candida* spp. activity was achieved by the agar–well diffusion method and the microdilution method for the determination of minimal inhibition concentration (MIC) and minimal fungicidal concentration (MFC) values. 

All *Candida* strains were adjusted to 10^7^ cells/mL (OD540 nm = 0.5) and then swabbed onto the surface of saboubouraud dextrose agar. Absorbent discs (Whatman disc number 3, 6 mm diameter) were impregnated with 10 μL of the tested EO, and then placed onto the surface of inoculated plates. Discs of standard antifungal agent itraconazole were taken as positive control. Following incubation for 24 h at 37 °C, the zone of inhibition (ZOI, in mm) was recorded, if present. All experiments were performed in triplicate [23].

#### 2.8.2. Minimum Inhibitory and Minimum Fungicidal Concentrations 

The minimum inhibitory concentration (MIC) values for *E. cardamomum* EO against all *Candida* strains were determined by the broth dilution method, according to the standard protocols [23]. The minimum inhibitory concentration (MIC) values for *E. cardamomum* EO against each strain were determined by the broth dilution method [23]. To determine the minimum fungicidal concentration (MFC) values, 10μL of each well medium with no visible growth was removed and plated into Sabouraud dextrose agar. After 24 h of incubation at 37 °C, the number of surviving organisms was determined as CFU/mL. MFC was defined as the lowest concentration at which 99% of the bacteria were killed. Itraconazole (12.5–0.003 mg/mL) was used as a positive control [24]. The fungicidal or fungistatic effect of green cardamom EO was determined according to the scheme proposed by Moroh et al. [25]. In fact, the tested EO was considered as fungistatic when the MFC/MIC ratio is greater than 4.

### 2.9. Effect of E. Cardamomum EO on Enzymatic Activities

Different *E. cardamomum* EO concentrations (MIC, 2× MIC and 4× MIC) were tested for their effect on phospholipase and proteinase activities against four *Candida* strains selected due to the result of the enzymatic properties after an initial characterization in Section 2.1 and Section 2.2. For this, *Candida* isolates incubated in Sabouraud broth combined with different *E. cardamomum* EO concentrations were cultured on phospholipase and proteinase agar media. Anti-phospholipase and proteinase activities were measured according to the methods described by Noumi et al. [16] by calculating the ratio between the diameter of the colony and that of the colony plus the precipitation zone.

### 2.10. Anti-Biofilm Activity of E. cardamomum EO

Determination of antibiofilm activity of the tested essential oil was carried out against *Candida* strains showing high biofilm formation ability. The reduction of biofilm growth and development by *E. cardamomum* EO was evaluated as previously described [26]. Biofilms were allowed to develop for 48 h at 37 °C in 96-well microtiter plates; this was followed by addition of different concentrations of *E. cardamomum* EO (1/2× MIC, 1× MIC, 2× MIC and 4× MIC). Then, 100 μL of the EO was dissolved in DMSO and Sabouraud broth was added to yield a concentration range of 1/2× MIC, 1× MIC, 2× MIC, and 4× MIC per well. The plates were further incubated for 24 h, which was followed by assessment of biofilms biomass by CV staining. CV-stained biofilm cells were quantified at 570 nm with the microplate reader, and the percentage of biofilm eradication was obtained by the following formula: ((OD growth control − OD sample)/OD growth control) × 100.

### 2.11. Statistical Analysis

All measurements were carried out in triplicate and the results were presented as mean values ± SD (standard deviation). Statistical analyses were performed using a one-way analysis of variance ANOVA test, and the significance of the difference between means was determined by Duncan’s multiple range test. Differences of *p* < 0.05 were considered statistically significant. 

## 3. Results

### 3.1. Enzymatic Characterization

**Figure 1:**
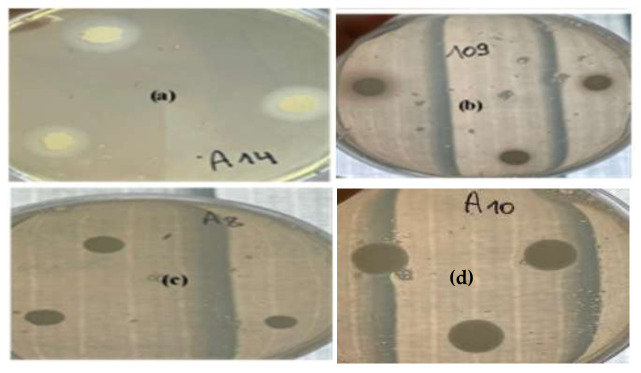
Appearance of positive (**a**) and negative (**d**) phospholipase activity on egg yolk agar; positive protease (**b**) and negative (**c**) activities on BSA agar.

The results of the distribution of the phospholipase and proteinase activities of the various *Candida* strains are presented in Table 1. Regarding phospholipase activity, 11 strains out of 20 (55%) had very high phospholipase activity (Pz < 0.69), which manifested as an opaque halo around the colonies on egg yolk agar (Figure 1a). Only four clinical strains (A10, 104, 108, and 118) were phospholipase-negative isolates (Table 1). Pz values, which are inversely proportional to the nature of the activity, ranged from 0.44 to 0.67 (Table 1). The activity of phospholipase (Pz values) was variable according to the species, with a maximum of 0.44 detected in the clinical strain of *C. albicans* (C4).

On the other hand, the incubation of *Candida* colonies for 7 days at 37 °C on BSA agar showed that only 4 strains out of 20 (20%) had very high protease activity (Pz < 0.69), as revealed by the presence of a clear halo around the colony on this medium (Figure 1a). Two reference strains (A1 and A15) had a high protease activity (Pz = 0.78 and 0.77 respectively). The reference *C. tropicalis* ATCC 1362 strain (A8) showed a very low protease activity (Figure 1c).

### 3.2. Chemical Composition of E. cardamomum EO 

The chemical composition of *E. cardamomum* EO is summarized in Table 2.

**Figure 2:**
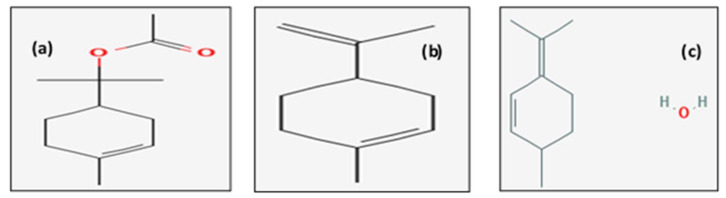
Chemical structure of the main compounds identified in *E. cardamomum* essential oil by GC-MS technique: (**a**) α-terpinyl acetate, (**b**) limonene, and (**c**) mentha-2,4(8)-diene.

Twenty-eight components with different percentage were identified using an HP5 capillary column according to their elution time. *E. cardamomum* EO was rich in α-terpinyl acetate (menthane monoterpenoids) (56.5%), limonene (cyclic monoterpene) (12.6%), and mentha-2,4(8)-diene (menthane monoterpenoids) (7.65%). Other relevant components were bornyl acetate (bicyclic monoterpenoids) (5.56%) and α-terpinene (monoterpene) (3.912%). The structures of the major compounds are represented in Figure 2. 

### 3.3. Antifungal Activity of E. cardamomum EO

The antifungal activity of the *E. cardamomum* EO against clinical and reference *Candida* strains is illustrated in Table 3. The results were based on the disk diffusion method, which is based on the determination of the zones of inhibition (ZI) on a solid medium and by the determination of the MICs and MFCs on a liquid medium.

The tested EO had greater activity against all tested *Candida* strains (two reference strains and seven clinical isolates) compared to the selected antifungal agent, itraconazole. Indeed, at a concentration of 10 mg/mL, the diameters of the zones of inhibition (ZI) measured on agar ranged from 7.33 ± 0.57 mm to 11.66 ± 0.57 mm. The largest inhibition diameter was recorded in the reference strain *C. utilis* ATCC 9255 (ZI = 11.66 ± 0.57 mm). The clinical isolate *C. albicans* (C5) was the most resistant strain (ZI = 7.33 ± 0.57 mm) to the activity of *E. cardamomum* EO at a concentration of 10 mg/mL (Table 3).

The results of MICs and MFCs obtained in our study ranged from 0.097 to 0.78 mg/mL and from 12.5 to 100 mg/mL, respectively. As there is no consensus on the acceptable level of inhibition for natural products compared to standard antibiotics, we considered the classification for plant materials proposed by Duarte et al. [27] based on the results of MICs: strong inhibitors, MIC up to 0.5 mg/mL; moderate inhibitors, MIC between 0.6 and 1.5 mg/mL; weak inhibitors, MIC greater than 1.6 mg/mL. Consequently, the data in Table 3 indicate that the tested EO can be considered a strong inhibitor against *Candida* strains (MIC= < 0.5 mg/mL). Using the scheme proposed by Moroh et al. [25], the tested EO showed greater fungistatic activity against all tested *Candida* strains (MFC/MIC ratio higher than 4) compared to the results of the same ratio obtained for the commercialized antifungal agent the itraconazole. These data are summarized in Table 3.

### 3.4. Anti-Enzymatic Activity of Green Cardamom EO 

The results of the study of the effect of different concentrations of *E. cardamomum* EO on hydrolytic enzymes are represented on Table 4. Our results demonstrated that the tested concentrations of green cardamom EO were not active against phospholipase produced by the reference strain of *C. albicans* ATCC 20402 (A15) and the clinical strain of *C. albicans* (113). In fact, the phospholipase activity was still very strong for *C. albicans* (A15) and strong for *C. albicans* (113). 

Concerning the second tested enzyme, the proteinase, the *C. albicans* (126) strain could produce this enzyme in the presence of MIC, 2xMIC, and 4xMIC of *E. cardamomum* EO. The clinical strain of *C. albicans* (109) could not produce either exoenzyme in the presence of the test EO, showing negative and very low activities for phospholipase and proteinase, respectively, compared to the control cases (Blank) (Table 4).

### 3.5. Adhesive Properties and Biofilm Formation 

**Figure 3:**
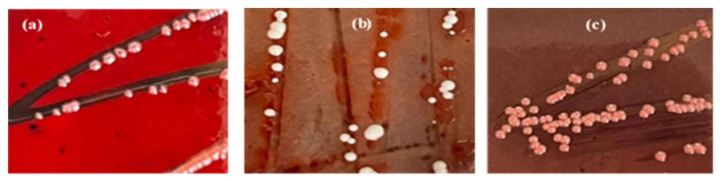
Different morphotypes of pathogenic strains cultivated on CRA: (**a**) positive morphotype; (**b**,**c**) negative morphotype.

Among tested strains, one out of twenty (5%) displayed variable phenotype (red with black center) over CRA plates (Figure 3), indicating slime production (Table 4).

The results of quantitative biofilm formation ability evaluated with CV staining assay revealed that among the tested strains, 8/24 (33.33%) of the strains were strong biofilm producers (OD_570_ ≥ 1) over polystyrene surfaces (Table 5). All the other strains showed moderate-grade biofilm formation (0.1 ≤ OD_570_ < 1) (Table 5).

**Figure 4:**
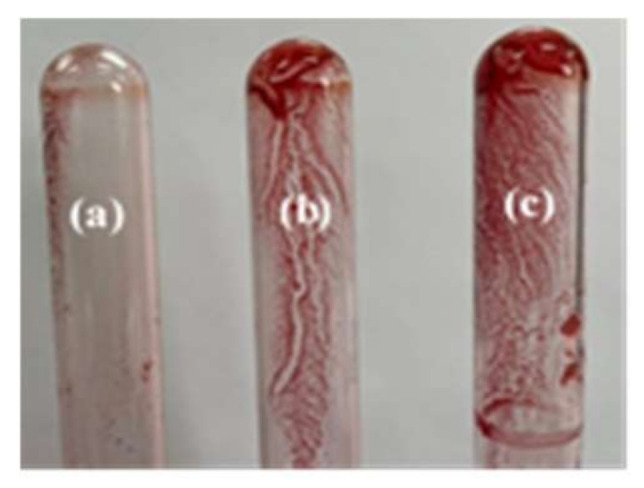
Adhesive properties on glass tube using safranin staining: (**a**) low adhesion (strain C4); (**b**) moderate adhesion (strain C3); (**c**) strong adhesion (strain 113).

The study of biofilm formation on glass tubes showed that six strains among twenty (30%) were strongly adherent (c, noted +++) and 50% (10 strains) were moderately adherent (b, noted ++) to this material (Figure 4).

### 3.6. Antibiofilm Activity of E. cardamomum EO 

Two reference strains of *Candida* (A1: *C. utilis* ATCC 9255 and A15: *C. albicans* ATCC 20402) and three clinical *C. albicans* isolates (C4, C5, and 113) were selected according to their strong biofilm production to study the antibiofilm ability of *E. cardamomum* EO.

At a concentration of 2× MIC, corresponding to 0.78 mg/mL against all *Candida* strains (except the strain C5), the EO exerted an anti-biofilm effect (OD570 < 1) when compared to the control (untreated cells) (Table 5). *E. cardamomum* EO was not effective against the clinical strain of *C. albicans* (C5) at 1/2× MIC, MIC, and 4× MIC (Table 6).

**Figure 5:**
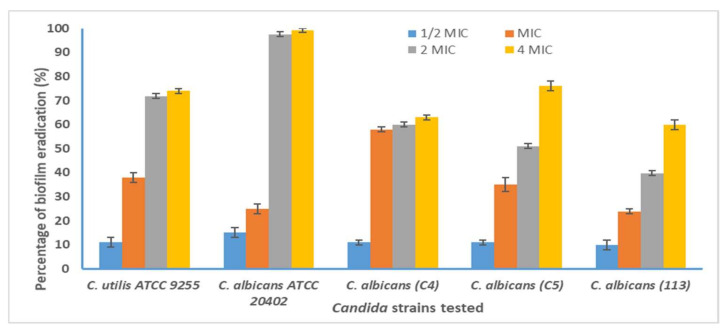
Anti-biofilm effect of the *E. cardamomum* EO against *Candida* strains using the crystal violet staining assay. Error bars represent standard deviations.

*E. cardamomum* EO showed anti-biofilm ability on clinical *C. albicans* strains (A15 and C5) on polystyrene of about 97.5% and 76%, respectively, at the highest tested concentration (4× MIC = 0.624 mg/mL) (Figure 5). The effect of *E. cardamomum* EO on candidal biofilm formed on polystyrene was variable depending on the concentration. A concentration equivalent to 4× MIC of the tested EO was able to inhibit the growth of the biofilm formed on polystyrene by all *Candida* strains with percentages of 60 ± 2% for *C. albicans* (113) to 99.2 ± 1 % for *C. albicans* ATCC 20402 (Figure 5).

## 4. Discussion

Fungal infections caused by *Candida* species are an important cause of mortality. Use of commercial treatment is limited due to the drug resistance, the toxicity of the antifungal agents, and drug–drug interactions [28]. Therefore, it is essential to develop new antifungal molecules with fewer risks and greater efficiency. Medicinal and aromatic plants have been of great importance for human health since ancient times. According to reports from the World Health Organization, 80% of the world’s population continues to use traditional medicines [29].

The *Candida* genus, particularly *C. albicans*, *C. tropicalis*, and *C. parapsilosis*, has been described as an important agent that is responsible for nosocomial infections [30,31]. 

Several virulence factors of this fungus have been extensively investigated, particularly regarding biofilm formation [32]. The results of quantitative biofilm formation investigation showed that all *Candida* isolates were biofilm producers on polystyrene surfaces but to different degrees. It has been previously demonstrated that *Candida* species are able to form biofilms on different abiotic surfaces. In fact, Vijayalakshmi [33] reported that *C. dubliniensis* (75%), followed by *C. glabrata* (67.9%), *C. albicans* (58.11%), *C. tropicalis* (53.33%), and *C. parapsilosis* (45.45%), were strong biofilm producers. Co-infection with other diseases can increase the biofilm-forming ability of *Candida* isolates [34].

*Candida* cells implicated into the biofilm are more resistant to azoles, especially fluconazole and clotrimazole [35]. In our case, a concentration of about 4× MIC of *E. cardamomum* EO showed anti-biofilm ability on all clinical *C. albicans* strains on polystyrene, to about 60% to 99.2%. This effect was variable depending on the concentration. 

The second virulence factor studied was exoenzyme production. In the current study, phospholipase production was reported in 80% of *Candida* isolates. In a previous study, 81.6% of *C. albicans* isolates showed phospholipase activity [36]. 

Concerning proteinase, the second exoenzyme, production was reported in 30% of the tested strains. Tsang et al. [37] reported that 82.1% of *C. albicans* strains were able to secrete the proteinase [37]. 

In our study, we studied the antifungal effect of *E. cardamomum* EO against the selected strains. Phytotherapy has used cardamom to treat cardiac and digestive disorders, tooth infections, diarrhea, and asthma [38,39,40]. This plant also exhibits antiulcerogenic [41] anti-inflammatory [42], antidiabetic [43], and antimutagenic [38] effects. 

Many researchers have demonstrated that *E. cardamomum* EO is rich in 1,8-cineol and poor in phenolic derivatives [44,45,46]. The chemical composition of the EO varies according to several factors, including harvesting region and time of plant collection. Some studies illustrated that this volatile oil contains 1,8-cineole (36.3%), α-terpinyl acetate (31.3%), limonene (11.6%), linalool (3%), and α-terpineol (2.6%) [47]. The EO of *E. cardamomum* was monoterpene-dominant. The main compound found in our *E. cardamomum* EO was α-terpinyl acetate (56.5%), followed by limonene (12.6%) and mentha-2,4(8)-diene (7.65%). Those three compounds represented more than 70% of the oil. Those results are in line with the literature, where terpinyl acetate is the most abundant component [48]. Still, there are other studies that refer to 1,8-cineol as the main compound [49]. It has been also reported that the cardamom aroma is due to the combination of the major components, 1,8-cineole and α-terpinyl acetate [50].

The antimicrobial properties of aromatic and medicinal plants are due to their chemical compounds, such as oils, alkaloids, tannins, and lipids [51,52]. *E. cardamomum* EO showed an effect when tested on *C. utilis* and *C. albicans* reference strains with an 11.66 mm and 10.66 mm zone of inhibition, respectively. This result was illustrated by Emira et al. [15] when they tested the effect of the same EO against *C. albicans* and *C. tropicalis* isolates [15]. The antibacterial activity may be the result of the presence of α-terpineol and linalool, compounds that are known to possess antibacterial activity [53]. In fact, terpineol and eugenol affected the morphology of *Staphylococcus aureus* and *Salmonella typhimurium*, indicating that the mechanism of action should be related by the rupture or dysfunction of the cell membrane [54].

At 10 µg/mL, the essential oil inhibits elastolytic and proteolytic activities in *Pseudomonas aeruginosa* PAO1. The 1,8-cineole can inhibit violacein production in *Chromobacterium violaceum*. Moreover, 1,8-cineole attenuated the expression of the tested quorum-sensing-controlled virulence factors (violacein pigment production, elastase and protease production, and motility) [15].

A previous study reported antimicrobial activity against *S. aureus* in acetonic extracts from *E. cardamomum* [26]. The same researchers demonstrated that the acetonic extract of cardamom was more active against *C. albicans* strains than the ethanolic extract [50]. *P. aeruginosa* was found to be the most resistant bacteria against the cardamom seed. The least inhibitory effects were observed for *K. pneumoniae*, *E. feacalis*, and *E. coli* [13].

The effects of natural substances (EO and plants extracts) on exoenzymes production have been largely described. In a previous study by Brondani et al. [55] the researchers demonstrated that exposure of sub-MICs to antimycotics significantly decreased proteinase production in all the *C. albicans* isolates tested. In the second study, Lyon et al. [56] reported that different surfactants did not affect phospholipase production. In *Origanum vulgare* essential oil, the enzymatic activity of *C. albicans* isolates decreased as the EO concentration increased, indicating the ability of the oil to inhibit such activity. In another study, Lima et al. [57] demonstrated the ability of some essential oils to inhibit the growth of some *Candida* species, including *C. albicans*, demonstrating possible disadvantages in the use of fungicides in relation to the use of anti-enzymatic compounds, especially anti-phospholipathics, which would reduce the virulence of the strains.

## 5. Conclusions

*Candida* species are normally found in human hosts and can survive in the hospital environment. The resistance of this fungus to commercialized antifungal agents has increased in recent years. Therefore, a new generation of more potent drugs needs to be developed to prevent the increasing threat of emerging azole-resistant *Candida* species. Modified medicines, such as plant materials and dry fruits, can be and are used for the treatment of these infections. 

Within the limitations of this study and based on the methodology used, it may be concluded that the essential oil of *Elettaria cardamomum* caused significant reductions in the virulence factors of *Candida* strains (biofilm formation and production of the phospholipase and proteinase enzymes). Thus, tests with green cardamom essential oil should be encouraged, since positive results for the decrease of the virulence of *C. albicans* with herbal medicines are of general interest, being able to produce medicines with fewer side effects to the users. This plant is recommended as an efficient alternative in medicine for the treatment of fungal infections and for biofilm inhibition.

## Figures and Tables

**Table 1:** Phospholipase and protease activities produced by *Candida* strains.

Strains	Species	Phospholipase		Protease	
		P_Z_* ± SD*	Activity	P_Z_* ± SD*	Activity
A1	*C. utilis* ATCC 9255	0.67 ± 0.02 ^a^	Strong	1 ± 0 ^a^	Very low
A4	*C. guillermondii* ATCC 6260	0.55 ± 0.005 ^a^	Very strong	0.78 ± 0.01 ^a^	High
A8	*C. tropicalis* ATCC 1362	0.49 ± 0.05 ^a^	Very strong	1 ± 0 ^a^	Very low
A14	*C. albicans* ATCC 10231	0.65 ± 0.017 ^ab^	Strong	1 ± 0 ^a^	Very low
A15	*C. albicans* ATCC 20402	0.45 ± 0.069 ^bc^	Very strong	0.77 ± 0 ^b^	High
A9	*S. cerevisiae* ATCC 20407	0.62 ± 0.03 ^cd^	Very strong	1 ± 0 ^b^	Very low
A10	*C. albicans*	1 ± 0 ^cd^	Negative	1 ± 0 ^b^	Very low
124	*C. albicans*	0.75 ± 0.02 ^cd^	Strong	1 ± 0 ^b^	Very low
C3	*C. albicans*	0.47 ± 0.05 ^cde^	Very strong	1 ± 0 ^c^	Very low
C2	*C. albicans*	0.58 ± 0.09 ^cde^	Very strong	0.84 ± 0.05 ^c^	Low
109	*C. albicans*	0.76 ± 0.04 ^cde^	Strong	0.69 ± 0.09 ^c^	Very high
113	*C. albicans*	0.6 ± 0.005 ^de^	Very strong	0.68 ± 0 ^c^	Very high
104	*C. albicans*	1 ± 0 ^e^	Negative	1 ± 0 ^c^	Very low
126	*C. albicans*	0.61 ± 0 ^f^	Very strong	0.65 ± 0.01 ^c^	Very high
104W	*C. albicans*	0.58 ± 0.02 ^fg^	Very strong	0.63 ± 0.12 ^c^	Very high
C4	*C. albicans*	0.44 ± 0.005 ^g^	Very strong	0.8 ± 0.05 ^c^	Low
108	*C. albicans*	1 ± 0 ^h^	Negative	1 ± 0 ^c^	Very low
118	*C. albicans*	1 ± 0 ^h^	Negative	1 ± 0 ^c^	Very low
C5	*C. albicans*	0.82 ± 0.03 ^h^	Strong	1 ± 0 ^c^	Very low
C1	*C. albicans*	0.62 ± 0.03 ^h^	Very strong	1 ± 0 ^c^	Very low

SD*: standard deviation; Pz* = 1 means that the isolate was phospholipase negative, Pz > 0.63 means that the strain was phospholipase positive, and when Pz < 0.63, the phospholipase activity was very strong. Pz between 0.9 and 1 indicates very low protease activity; 0.89–0.80, low proteinase activity; 0.79–0.70, high protease activity, and Pz < 0.69, very high protease activity. The letters (a–h) indicate a significant difference according to Duncan’s test (*p* < 0.05).

**Table 2:** Chemical composition of *Elattaria cardamomum* essential oil by GC-EIMS.

N	Compound Name	RT*	Amount	Ki*	Kr*
1	Camphene	13.674	0.135	869.4876	954
2	Δ-2-carene	15.293	0.098	890.4374	1002
3	α-phellandrene	17.682	0.856	921.3509	1002
4	**α-terpinene**	18.812	**3.912**	935.9731	1017
5	**Limonene**	19.864	**12.598**	949.5859	1029
6	γ-terpinene	21.7	1.65	973.3437	1059
7	Mentha-3,8-diene <p->	22.444	0.265	982.971	1072
8	**Mentha-2,4(8)-diene <p->**	23.953	**7.649**	999.0217	1088
9	Menthatriene <1,3,8-p->	25.348	0.166	1017.716	1110
10	Camphor	27.288	0.38	1043.715	1146
11	Limonene-1,2-epoxide (Fr.1)	30.241	0.151	1083.289	/
12	α-terpineol	31.094	0.227	1094.72	1188
13	Fenchyl Acetate	32.904	0.539	1113.468	1220
14	Mentha-1(7),8-dien-2-ol <cis-p->	33.596	0.379	1123.372	1230
15	Perilla Aldehyde	35.006	0.451	1143.552	1271
16	Isopulegol Acetate	37.237	0.265	1175.483	1277
17	**Bornyl Acetate**	37.625	**5.561**	1181.036	1288
18	Terpinen-4-ol Acetate	38.427	0.942	1192.515	1299
19	Pinocarvyl Acetate <cis->	40.126	0.388	1211.981	1312
20	**α-Terpinyl Acetate**	43.06	**56.499**	1256.422	1349
21	6,8-nonadien-2-one-6-methyl-5-(1-methylethylidene)-	43.22	0.169	1258.846	/
22	Carvone Hydrate	45.133	1.728	1287.822	1423
23	Cytronellyl Propanoate	46.602	0.395	1304.196	1446
24	Trans-p-mentha-2,8-dienol	47.06	1.393	1311.586	/
25	Limonene-1,2-epoxide (Fr.1)	49.676	0.454	1353.8	/
26	Pulegone	51.84	0.214	1388.72	1237
27	Hydroxy-α-terpenyl Acetate	52.549	0.987	1395.047	/
28	Carvone Acetate	54.878	0.91	1434.555	/

RT*: retention time; Ki*: Kovats retention index determined relative to the tR of a series of n-alkanes (C10–C35) on an HP-5 MS column; Kr*: Kovats retention index determined relative to the tR of a series of n-alkanes (C10–C35) on HP Innowax.

**Table 3:** Antifungal activity and MFC/MIC ratio values of *E. cardamomum* EO and itraconazole against reference and clinical *Candida* strains.

Strains	Species	*Elattaria cardamomum* EO (10 mg/mL)			MFC/MIC Ratio	Itraconazole (10 mg/mL)			MFC/MIC Ratio
		IZ* ± SD* (mm)	MIC* (mg/mL)	MFC* (mg/mL)		IZ ± SD (mm)	MIC (mg/mL)	MFC (mg/mL)	
A1	*C. utilis* ATCC 9255	11.66 ± 0.57 ^a^	0.78	100	>4;Fungistatic	6 ± 0 ^a^	0.156	2.5	>4;Fungistatic
A15	*C. albicans* ATCC 20402	10.66 ± 0.57 ^ab^	0.156	100	>4;Fungistatic	14.66 ± 0.57 ^a^	0.078	10	>4;Fungistatic
109	*C. albicans*	8 ± 0 ^bc^	0.097	100	>4;Fungistatic	7.33 ± 0.57 ^a^	0.156	2.5	>4;Fungistatic
113	*C. albicans*	8.66 ± 0.57 ^cd^	0.156	100	>4;Fungistatic	6 ± 0 ^b^	0.312	10	>4;Fungistatic
126	*C. albicans*	9.66 ± 0.57 ^d^	0.126	12.5	>4;Fungistatic	11.66 ± 0.57 ^b^	0.312	10	>4;Fungistatic
104W	*C. albicans*	9.66 ± 0.57 ^d^	0.156	50	>4;Fungistatic	10.33 ± 0.57 ^c^	0.312	10	>4;Fungistatic
C4	*C. albicans*	9.66 ± 0.57 ^d^	0.156	100	>4;Fungistatic	6 ± 0 ^c^	0.312	10	>4;Fungistatic
C5	*C. albicans*	7.33 ± 0.57 ^e^	0.156	25	>4;Fungistatic	7.33 ± 0.57 ^d^	0.078	10	>4;Fungistatic
C1	*C. albicans*	9 ± 0 ^f^	0.156	50	>4;Fungistatic	10.33 ± 0.57 ^e^	0.312	10	>4;Fungistatic

IZ*: inhibition zone; SD*: standard deviation; MIC*: minimal inhibition concentration; MFC*: minimal fungicidal concentration; EO: essential oil. The letters (a–f) indicate a significant difference according to Duncan’s test (*p* < 0.05).

**Table 4:** Effect of different concentrations of *E. cardamomum* EO on exoenzyme production in *Candida* strains.

Strains	Concentration	Phospholipase		Proteinase	
		OD*_570 nm_ ± SD*	Activity	OD*_570 nm_ ± SD*	Activity
A15	Blank*	0.45 ± 0.069	Very strong	0.77 ± 0	High
	**MIC**	0.49 ± 0.05	Very strong	1 ± 0	Very low
	**2xMIC**	0.58 ± 0.09	Very strong	1 ± 0	Very low
	**4xMIC**	0.62 ± 0.03	Very strong	1 ± 0	Very low
109	**Blank**	0.76 ± 0.04	Strong	0.69 ± 0.09	Very high
	**MIC**	1 ± 0	Negative	0.82 ± 0.03	Low
	**2xMIC**	1 ± 0	Negative	1 ± 0	Very low
	**4xMIC**	1 ± 0	Negative	1 ± 0	Very low
113	**Blank**	0.6 ± 0.005	Very strong	0.68 ± 0	Very high
	**MIC**	0.67 ± 0.03	Strong	0.87 ± 0.03	Low
	**2xMIC**	0.69 ± 0.04	Strong	1 ± 0	Very low
	**4xMIC**	0.73 ± 0.03	Strong	1 ± 0	Very low
126	**Blank**	0.61 ± 0	Very strong	0.65 ± 0.01	Very high
	**MIC**	0.9 ± 0	Strong	0.67 ± 0.02	Very high
	**2xMIC**	1 ± 0	Negative	0.75 ± 0.04	High
	**4xMIC**	1 ± 0	Negative	0.7 ± 0.03	High

OD*: optical density; SD*: standard deviation; MIC*: minimal inhibitory concentration; Blank*: untreated cells with EO.

**Table 5:** Slime production and qualitative and quantitative adhesive properties of *Candida* strains on glass and polystyrene.

Strains	Adhesion to Glass	Slime on CRA*		Biofilm on Polystyrene	
		Morphotype	S+*/S-*	OD*± SD*	Biofilm Ability
A1	**+**	White	S-	1.81 ± 0.05 ^a^	Strong biofilm
A4	**++**	Pink	S-	0.34 ± 0.07 ^a^	Moderate biofilm
A8	**+++**	Red	S-	0.81 ± 0.05 ^b^	Moderate biofilm
A9	++	Red	S-	0.7 ± 0.01 ^b^	Moderate biofilm
A10	**+++**	Pink	S-	0.43 ± 0.03 ^bc^	Moderate biofilm
A14	**++**	Red	S-	0.77 ± 0.03 ^bc^	Moderate biofilm
A15	++	Red	S-	1.07 ± 0.06 ^cd^	Strong biofilm
124	**+++**	Red with black center	S+	0.56 ± 0.09 ^cde^	Moderate biofilm
C3	**++**	Pink	S-	1.55 ± 0.17 ^def^	Strong biofilm
C2	**++**	Pink	S-	0.55 ± 0.09 ^ef^	Moderate biofilm
109	**++**	Pink	S-	0.66 ± 0.1 ^f^	Moderate biofilm
113	**+++**	Pink	S-	1.18 ± 0.06 ^f^	Strong biofilm
104	**+++**	Pink	S-	0.66 ± 0.1 ^g^	Moderate biofilm
126	**+++**	Red	S-	0.92 ± 0.04 ^h^	Moderate biofilm
104W	**++**	Pink	S-	0.82 ± 0.04 ^i^	Moderate biofilm
C4	**+**	White	S-	1.66 ± 0.1 ^j^	Strong biofilm
108	**+**	White	S-	0.92 ± 0.04 ^j^	Moderate biofilm
118	**+**	Pink	S-	2.17 ± 0.2 ^k^	Strong biofilm
C5	**++**	Bordeaux	S-	1.68 ± 0.2 ^l^	Strong biofilm
C1	**++**	White	S-	2.33 ± 0.11 ^m^	Strong biofilm

CRA*: Congo red agar; S+*: slime-positive: S-*: slime-negative; OD*: optical density; SD*: standard deviation. The letters (a–m) indicate a significant difference according to Duncan’s test (*p* < 0.05). Weak slime production (+), moderate slime production (++), or strong slime production (+++).

**Table 6:** Effect of 1/2× MIC, MIC, 2× MIC, and 4× MIC of *E. cardamomum* EO against *Candida* strains biofilm formed on polystyrene.

Strains	Concentration	OD*_570 nm_ ± SD*	Biofilm Ability
A1	Blank*	0.81 ± 0.05 ^a^	Strong biofilm
	1/2× MIC*	0.7 ± 0.01 ^a^	Moderate biofilm
	**MIC**	0.43 ± 0.03 ^b^	Moderate biofilm
	**2× MIC**	0.092 ± 0.004 ^c^	Low biofilm
	**4× MIC**	0.07 ± 0.009 ^d^	Low biofilm
A15	**Blank**	1.07 ± 0.06 ^a^	Strong biofilm
	**1/2× MIC**	0.92 ± 0.04 ^a^	Moderate biofilm
	**MIC**	0.82 ± 0.04 ^b^	Moderate biofilm
	**2× MIC**	0.095 ± 0.004 ^c^	Low biofilm
	**4× MIC**	0.078 ± 0.008 ^d^	Low biofilm
C4	**Blank**	0.66 ± 0.1 ^a^	Strong biofilm
	**1/2× MIC**	0.55 ± 0.09 ^a^	Moderate biofilm
	**MIC**	0.08 ± 0 ^a^	Low biofilm
	**2× MIC**	0.06 ± 0.01 ^b^	Low biofilm
	**4× MIC**	0.03 ± 0.02 ^c^	Low biofilm
C5	**Blank**	1.68 ± 0.2 ^a^	Strong biofilm
	**1/2× MIC**	1.57 ± 0.19 ^ab^	Strong biofilm
	**MIC**	1.33 ± 0.17 ^bc^	Strong biofilm
	**2× MIC**	1.17 ± 0.19 ^bd^	Strong biofilm
	**4× MIC**	0.92 ± 0.18 ^d^	Moderate biofilm
113	**Blank**	1.18 ± 0.06 ^a^	Strong biofilm
	**1/2× MIC**	1.08 ± 0.01 ^b^	Strong biofilm
	**MIC**	0.942 ± 0.05 ^c^	Moderate biofilm
	**2× MIC**	0.782 ± 0.04 ^d^	Moderate biofilm
	**4× MIC**	0.58 ± 0.03 ^e^	Moderate biofilm

OD*: optical density; SD*: standard deviation; MIC*: minimal inhibitory concentration. The letters (a–e) indicate a significant difference according to Duncan’s test (*p* < 0.05); Blank*: untreated cells with EO.

## Data Availability

Not applicable.
